# Residential proximity to metal emitting industries and toenail metal concentration in the US Gulf States

**DOI:** 10.21203/rs.3.rs-3210942/v1

**Published:** 2023-08-07

**Authors:** Joyce JY Lin, Emily Werder, Kaitlyn G Lawrence, W. Braxton Jackson, Dale P Sandler, Aisha S Dickerson, Lawrence S Engel, Ana M Rule

**Affiliations:** Johns Hopkins University Bloomberg School of Public Health; National Institute of Environmental Health Sciences Laboratory of Pharmacology and Chemistry: National Institute of Environmental Health Sciences; National Institute of Environmental Health Sciences; Social & Scientific Systems Inc, a DLH Holdings Corp; National Institute of Environmental Health Sciences; Johns Hopkins University Bloomberg School of Public Health; The University of North Carolina at Chapel Hill Gillings School of Global Public Health; Johns Hopkins University Bloomberg School of Public Health

**Keywords:** Metals, Exposure Assessment, Environmental Justice, Toenail Biomarkers

## Abstract

**Objective:**

The US Gulf region is heavily reliant on metal-emitting petrochemical and manufacturing industries. We characterized the effect of residential proximity to metal-emitting sites and metal body burden in Gulf states residents with particular attention to potential differential exposure burden by race.

**Methods:**

We measured toenail concentrations of arsenic, chromium, lead, manganese, mercury, and selenium using inductively coupled plasma mass spectrometry in 413 non-smoking men from the Gulf Long-term Follow-Up Study. Point sources of industrial metal emissions were identified using the US EPA’s National Emissions Inventory (NEI) database and geocoded to participant residential addresses. For each metal, we assessed associations of toenail metal concentrations with the inverse-distance weighted number of emissions sites and volume of air-metal emissions within 30 km radial buffers of participant residences using multivariable linear regression. Results were stratified by race.

**Results:**

Compared to self-identified Non-Hispanic (NH) White participants, NH Black participants lived closer to NEI sites but had 23-70% lower toenail metal concentrations adjusting for other personal/behavioral factors. Residential proximity to lead-emitting NEI sites was positively associated with toenail Pb concentration while proximity to mercury-emitting NEI sites was inversely associated with toenail Hg concentration. Findings for lead were significantly attenuated after adjustment for neighborhood-level socioeconomic factors.

**Conclusion:**

Residential proximity to lead-emitting NEI sites in the US Gulf region is associated with a higher body burden of lead. However, this relationship may be driven in part by non-NEI factors related to residence in industry-adjacent neighborhoods.

## 1. Introduction

Pollutant metals and metalloids, hereafter referred to as “metals", exist ubiquitously in the environment but concentrate in certain areas as a result of anthropogenic emissions from industry, agriculture, fossil fuel combustion, and waste disposal (Tchounwou et al., 2012). Since these elements do not degrade, their accumulation in the environment greatly increases the risk of chronic human exposure. While the degree of metal toxicity is determined by the chemical type, dose, and route of exposure, a wealth of evidence points to numerous adverse health effects associated with a wide range of metal exposures across the life course (Attreed et al., 2017; Jusko et al., 2008; Vahter et al., 2002; Wright & Baccarelli, 2007). Given well-documented effects of metal toxicity, the rapidly evolving commercial uses for metals in industrial processes have raised concerns about chronic metal exposure for communities residing proximal to industrial areas (Tchounwou et al., 2012).

The concentration of industrial operations in the US Gulf of Mexico region presents a serious risk of chronic ambient metal exposure. Metal byproducts from large-scale chemical, plastics, paper, and electrical manufacturing constitute some of the largest point sources of ambient environmental metal exposure in the Gulf region (Ha et al., 2014; Hassaan et al., n.d.). In 2014, across Louisiana, Alabama, Mississippi, and Florida, more than 23 types of industries released more than 129 million pounds of various metals directly into the air, soil, and water. This concentration of industries, coupled with the Gulf region’s extensive history of racial segregation raises concerns of disproportionate metal exposures which may further exacerbate existing health disparities in the region. While historically marginalized and low-income communities have repeatedly been shown to bear disproportionate burdens of environmental pollution in the US (Apelberg et al., 2005; Hajat et al., 2015; Jones et al., 2022; Trottier et al., n.d.), few studies have assessed the impact of industrial emissions on human metal exposure using biomonitoring. To our knowledge, no such studies have been conducted in the uniquely vulnerble US Gulf region.

In this study, we examine the relationship between residential proximity to industry-reported air metal emissions and toenail metal concentration in a multi-state sample of men from the Gulf Long-term Follow up (GuLF) Study. Analyses were stratified by self-reported race in consideration of potential metal exposure disparities related to the persistent effects of the area’s extensive history of racial segregation.

## 2. Methods

### 2.1 Study population

The Gulf Long-term Follow-up (GuLF) Study (2011–2013) is a large prospective cohort study (33,608) of short and long-term health effects related to oil spill exposures from the 2010 *Deepwater Horizon* (DWH) disaster (Kwok et al., 2017). Participants comprise individuals who either worked on the oil spill for at least one day (oil spill cleanup workers) or who took part in mandatory worker safety training but did not work on the spill (non-workers). Details about GuLF Study enrollment and cohort follow-up have been previously published (Engel et al., 2017; Kwok et al., 2017).

**Figure 1:**
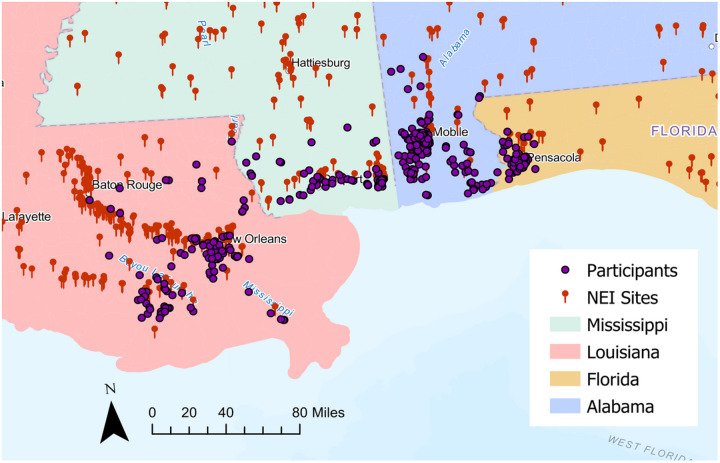
Geographic distribution of study participants relative to metal emitting NEI sites (n=413)

This research was conducted in a sample of 413 non-smoking men from the GuLF Study who provided toenail samples at a clinical exam visit 2 to 6 years (median 4.6 years) after the end of reported cleanup from the *DWH* disaster. Detailed selection criteria for the subsample in this study have been reported previously (Lin et al., 2023). Briefly, between August 2014 and June 2016, 3,401 individuals who lived within 60 miles of study clinics in Mobile, Alabama, or New Orleans, Louisiana participated in a clinical exam (CE) in which trained examiners collected health, diet, work history, and residential address, as well as toenail biospecimens, anthropometric measures and neurobehavioral test results. The geographic distribution of participants in the analytic sample is shown in Fig. 1. Toenail samples were collected in paper envelopes and stored at room temperature in the GuLF Study biorepository until analysis in 2021. Of participants who completed a neurobehavioral exam and provided sufficient toenail samples (n = 2,734), we included those with previously measured liver and kidney function/injury biomarkers, selected on the basis of oil spill exposures (N = 679), to maximize GuLF Study biomarker overlap. We further excluded self-reported current smokers to focus our analysis. This resulted in a final analytical population of 413 participants eligible for inclusion in this study.

### 2.2 Toenail Metals Analysis

Toenail samples were analyzed for 18 metals/metalloids (aluminum (Al), antimony (Sb), arsenic (As), cadmium (Cd), calcium (Ca), chromium (Cr), cobalt (Co), copper (Cu), iron (Fe), lead (Pb), magnesium (Mg), manganese (Mn), mercury (Hg), molybdenum (Mo), nickel (Ni), selenium (Se), vanadium (V), and zinc (Zn)) using inductively coupled plasma mass spectrometry (ICP-MS). Roughly 1–2 toenail clippings (median 25 mg) were randomly selected from each participant’s total sample for metals analysis. This sub-sampling method maximizes sample conservation and has demonstrated reliable results in previous findings (Lin et al., 2023).

Details about the toenail metal analysis process are described elsewhere (Lin et al., 2023). In brief, toenail samples were cleaned using a multi-stage wash process involving 30% acetone, 1% Triton X-100 solution, and Mili-Q water. Cleaned toenail samples were digested using an open vessel microwave assisted digestion method adapted from the Dartmouth Trace Element Analysis Core (Andrew et al., 2020). Briefly, samples were digested with 0.5 ml nitric acid (HNO_3_) and 0.2 ml of hydrogen peroxide (H_2_O_2_) optima and heated to 110°C before being diluted with 0.5% hydrochloric acid (HCl) for analysis by ICP-MS (Agilent 8800 ICP-MS Triple Quad; Agilent technologies, Inc., Santa Clara, CA). Data quality was monitored via multipoint calibration curves for each analyte at the beginning and end of each batch, analysis of laboratory and digestion blanks, duplicates, spikes, and comparison with two reference materials: human hair Japan NIES #13 (National Institute for Environmental Studies, Ibaraki, Japan) and caprine horn NYS RM 1801 (New York State Department of Health Wadsworth Center, Albany, NY). The average between-batch coefficient of variation across metals was 11% and ranged from 3% (Pb) to 27% (Sb). The limit of detection (LOD) for each metal was calculated using 3 times the standard deviation of digestion blanks (N = 7) for each batch. The average LOD for each metal across batches ranged from 0.00002 μg/g for Cd to 0.3 μg/g for Ca (Supplemental Table 1).

This analysis focused on elements that were both detected in > 85% of toenail samples and reported by the National Emissions Inventory (NEI) (As, Cr, Hg, Mn, Pb, Se). Ni was excluded despite high detection (100%) and NEI reporting because our previous reliability study found no correlation between toenail Ni concentrations from the same person over two time points ~ 3 years apart, suggesting that the toenail matrix may not be a good biomarker of chronic Ni exposure (Lin et al., 2023). Samples below the LOD (As, n = 54; Cr, n = 29; Pb, n = 25; Mn, n = 8; Hg, n = 25, Se, n = 4) were assigned a value of the batch-specific LOD divided by √2 (Helsel, 2005).

### 2.3 National Emissions inventory

Sources of anthropogenic metal emissions were identified using the National Emissions Inventory (NEI), the US Environmental Protection Agency’s (EPA) most comprehensive database of annual criteria, precursor, and hazardous air pollutant emissions (*National Emissions Inventory 2014*, 2017). Estimates provided by the NEI are compiled using reporting data provided by State, Local, and Tribal air agencies that are supplemented by information from the Toxics Release Inventory, the Acid Rain Program, and EPA’s regulatory air toxics data. We abstracted all records of reported emissions of available metals (As, Cr, Hg, Mn, Pb, Se) from the 2014 NEI point source database and geocoded participant residential addresses relative to the locations of NEI sources to assess potential associations between residential NEI proximity and toenail metal concentration.

We assigned exposure to point sources of metal air emissions using 3 methods. First, we assessed the linear distance from the residence to the nearest metal emitting site (Distance km). Second, we calculated the sum of the inverse distance weighted number of sites within 30 km of the participants’ clinical exam residences (Site IDW). Third, we calculated the sum of the inverse distance weighted pounds of emissions within 30 km of the participants’ residences (Emissions IDW).

### 2.4 Statistical Methods

We used multivariable linear regression to estimate the difference in the log10-transformed toenail metal concentrations and 95% confidence interval (CI) per unit increase in each of the NEI proximity metrics (Distance km, Site IDW, and Emissions IDW scores). Models were adjusted for individual-level physiological or behavioral factors that could influence toenail metal concentration including age, cigarette smoking history, body mass index (BMI), passive smoke exposure (> 30 mins of smoke exposure per day on average), employment status (working, unemployed/retired), and state of residence. All individual-level covariate data were ascertained from the GuLF Study clinical exam and follow-up questionnaires closest to the time of toenail collection.

Analyses were stratified by self-reported race and income to assess potential disparities related to the effects of historic segregation and persistent racism in this area. Given the small proportion of participants in other racial categories, analyses of racial disparities focused on comparisons between the Non-Hispanic (NH) White and NH Black participant groups.

In secondary analyses, we additionally adjusted for social factors such as individual level of educational attainment and neighborhood level variables (median household income, percent of households below the poverty level, and median year that structures were built) using data from the 2014 American Community Survey (ACS) at the census block group level to address potential influence from unmeasured confounders related to residential proximity to industry. Social variables were each included in separate models to reduce the impact of collinearity. Beta estimates for all models were converted to percent differences using the formula (10^*β*^ – 1) * 100

### 2.5 Sensitivity Analyses

Toenail samples analyzed in this study were collected 2–6 years (median: 4.6) after the end of self-reported oil spill cleanup work and thus are well beyond the expected exposure window relevant to oil spill cleanup exposure. However, given the occupational origins of this cohort, we conducted sensitivity analyses including cleanup-related cumulative total average hydrocarbon (Cumulative THC; ppm) inhalation exposure estimates as a proxy of oil spill cleanup involvement and intensity in our models.

## 3. Results

### 3.1 Participant characteristics

Forty-six percent (n = 191) of participants in this study self-identified as NH Black, 46% (n = 190) identified as NH White, and 8% (n = 32) identified as Asian, American Indian/Alaskan Native, Mixed Race, or Other. On average, White participants were older, had higher educational attainment, higher annual household income, were more frequently former smokers, and currently employed compared to Black participants. There were also differences in racial makeup by state, with the majority (53%) of Black participants residing in Alabama and White participants having more even distribution across the 4 states (Table 1).

Seventy-eight percent of all participants lived within 10 km of a metal emitting NEI site. Ninety-six percent of Black participants lived within 10 km of a metal emitting NEI site, 79% within 5 km, and 49% within 3 km of a metal emitting NEI site compared to 63%, 32%, and 17% of White participants within 10 km, 5 km, and 3 km, respectively. Racial differences in residential proximity to metal emitting NEI sites persisted after accounting for income, with Black participants living closer to metal emitting NEI sites than White participants within every income category (Supplemental Fig. 1).

### 3.2 Industrial determinants

We observed significant associations between NEI sources of metal exposure and toenail metal concentrations for Pb and Hg. No significant associations with NEI sites were observed for any of the remaining metals tested (As, Cr, Mn, and Se). Other personal and behavioral factors associated with toenail Pb and Hg concentrations are provided in Supplemental Table 1. Oil spill cleanup involvement was not related to toenail metal concentrations of any of the elements of interest in this study.

#### 3.2.1 Lead (Pb)

**Figure 2:**
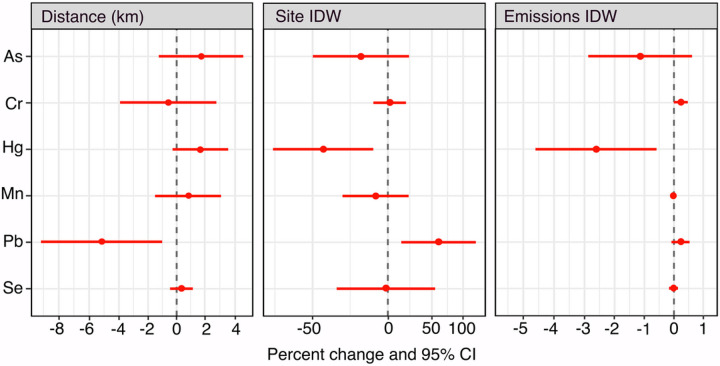
Relationship between industrial determinants of metal exposure (Distance, Site IDW, Emissions IDW) and toenail metal concentrations adjusted for age, cigarette smoking history, body mass index (BMI), passive smoke exposure (>30 mins of smoke exposure per day on average), employment status (working, unemployed/retired), and state of residence. Note: Distance (km) = residential distance from the closest NEI site. Site IDW = inverse distance weighted number of sites within 30 km of residence. Emissions IDW = inverse distance weighted volume of emissions within 30 km of residence.

Distance to the nearest Pb emitting NEI site and density of NEI sites within 30 km of the residence (measured by the Site IDW score) were positively associated with toenail Pb concentration. For every 1 km increase in the distance from the home to the nearest Pb NEI site, we observed – 5.10% (95% CI: −9.07, −0.95) change in toenail Pb concentration after adjusting for personal and behavioral characteristics such as age, BMI, smoking history, passive smoke exposure and employment status. Similarly, for every 1 unit increase in Site IDW score (higher NEI density around the home), we observed 64.49% (95% CI: 16.64, 131.96) higher toenail Pb concentrations after adjusting for personal and behavioral characteristics. Emissions volume from nearby NEI sites (measured by the Emissions IDW score) was positively related to toenail Pb concentration but the association was not statistically significant (Fig. 2).

#### 3.2.2 Mercury (Hg)

Unexpectedly, proximity to Hg emitting NEI sites was inversely associated with toenail Hg concentration. For every 1 unit increase in Site IDW score (higher density), toenail Hg concentrations changed by – 47.35% (95% CI: −66.85, −16.40). We observed a similar trend with emissions volume. For every unit increase in Emissions IDW score (more emissions), we observed – 2.62% (95% CI: −4.61, −0.58) changes in toenail Hg concentration (Fig. 2). Distance to the nearest Hg NEI site was not significantly associated with toenail Hg concentration.

### 3.3 Stratification by race

Given the racial differences in residential proximity to NEI sites in this study, we stratified analyses by race to examine potential inequities in metal exposure burden from metal-emitting NEI sites. The association between Pb Site IDW score and toenail Pb was stronger in Black participants (109.95% (95% CI: 17.52, 275.07) compared to White participants (52.45% (95% CI: −11.64, 163.03). The associations for Hg Site and Emissions IDW scores were similarly driven by stronger associations among Black participants. For every 1 unit increase in Hg Site IDW score, we observed – 72.13% (95% CI: −88.48, −32.54) change in toenail Hg among Black participants and – 3.52% (95% CI: −47.10, 75.97) among White participants. Similarly, for every 1 unit increase in Hg Emissions IDW score, we observed – 4.70% (95% CI: −8.17, −1.09) change in toenail Hg among Black participants compared to −0.81 % (95% CI: −3.29, 1.73) among White participants (Supplemental Fig. 2).

### 3.4 Secondary analysis

Since industrial sites tend to concentrate in disadvantaged neighborhoods that may also experience disproportionate exposures to metals through other non-industrial sources, the additional adjustment for individual level and neighborhood level socio-economic variables allowed us to understand the extent to which observed associations could be reasonably attributed to metal exposures from unmeasured non-NEI sources.

**Figure 3:**
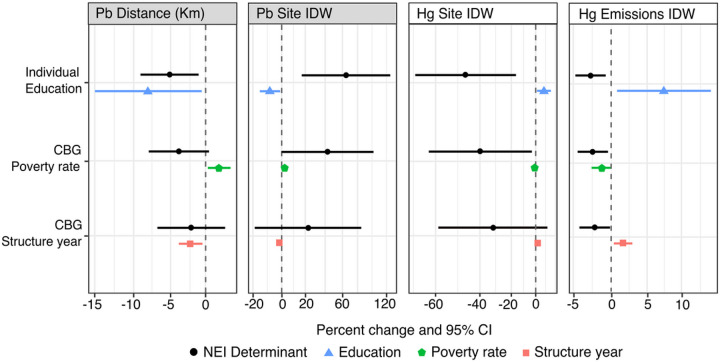
Industrial determinants additionally adjusted for individual and neighborhood level SES variables (each in separate model)

After additionally adjusting for individual-level educational attainment, distance from the nearest Pb-emitting NEI site and density of Pb NEI sites within 30 km of the residence remained significantly associated with toenail Pb concentration at −5.10% (95% CI: −9.07, −0.95) and 64.49% (95% CI: 16.64, 131.96), respectively. After adjusting for neighborhood-level social factors including the percent of residents living below the poverty line and the median year that structures are built within the census block group, the association between toenail Pb and distance/density of Pb NEI sites remained in the expected direction but the effect was significantly attenuated (Fig. 3). Greater attenuation was observed after adjusting for the census block group median structure year (−2.07% (95% CI: −6.83, 2.92) than after adjusting for census block group percent below poverty level (−3.83 (95% CI: −8.00, 0.53). The same trend was observed for Pb Site IDW (Fig. 3).

Site and Emissions IDW associations for Hg remained after adjustment for individual level of education but were also attenuated after adjustment for neighborhood-level SES factors such as census block poverty rate and median year that structures were built (Fig. 3). Unlike for Pb, the attenuated inverse associations between Site IDW score and toenail Hg concentration remained statistically significant after adjustment for census block group poverty rate (−39.77% (95% CI: −62.48, −3.32). The Emissions IDW score variable for Hg also remained statistically significant after adjusting for census block group poverty rate (−2.45% (95% CI: −4.45, −0.41)) and the median year that structures were built within the census block group (−2.20% (95% CI: −4.21, −0.14)).

### 3.5 Differences in toenail metal concentration by race

**Figure 4:**
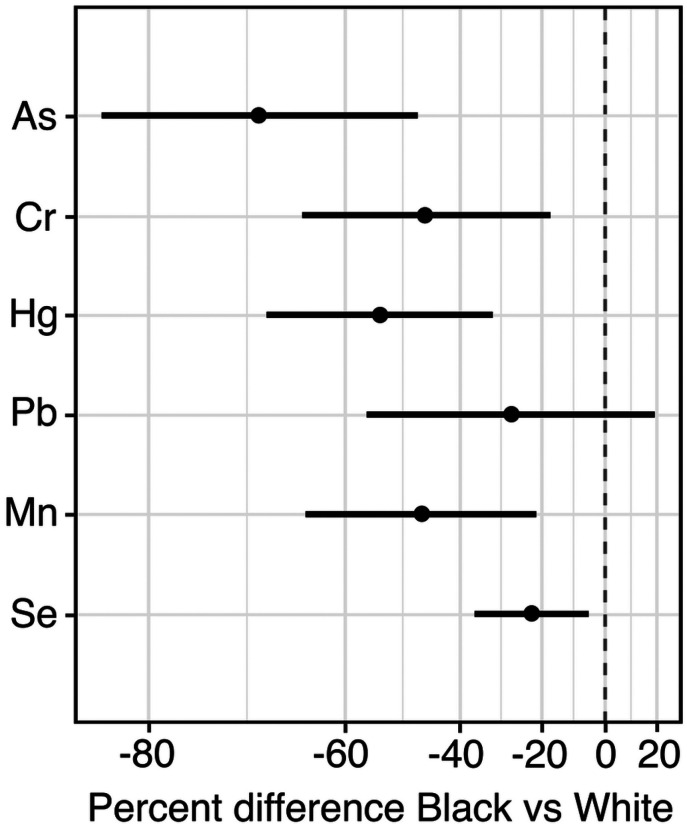
Differences in toenail metal concentrations by race

Despite the proximity of Black residences near metal-emitting NEI sites, concentrations of all toenail metals tested except for Pb were significantly lower among Black participants (As (−70.41 % (95% CI: −83.08, −48.26)), Cr (−46.76% (95% CI: −65.68, −17.39)), Hg (−54.84% (95% CI: −69.73, −32.63)), Mn (−47.77% (95% CI: −65.27, −21.46)), and Se% (−22.83 (95% CI: −36.94, −5.57)) compared to White participants after adjusting for personal and environmental factors such as age, BMI, smoking history, passive smoke exposure, work status and state of residence. Median toenail Pb concentration was also lower among Black participants (−28.30% (95% CI: −56.92, 19.32), but the difference was not statistically significant (Fig. 4).

We also examined differences in toenail metal concentration by income group and found that participants making less than or equal to $20,000 a year had toenail Pb concentrations that were 121.21% (95% CI: 18.70, 312.25) higher than those reporting making more than $50,000 a year. Significant differences in toenail metal concentration were also observed for Cr and Hg with those making less than $20,000 a year having toenail Cr and Hg concentrations that were 45.73% (95% CI: 6.82, 68.39) and 64.66% (95% CI: 41.80, 78.54) lower, respectively, than those making more than $50,000 a year (Supplemental Fig. 5).

## 4. Discussion

In this multi-state study of industrial metal exposures in the US Gulf, we found differences in residential proximity to NEI sites by self-reported race and significant associations between residential proximity to NEI sites and toenail Hg/Pb concentrations. Higher density of Pb-emitting NEI sites within 30 km of the residence (Site IDW) was positively associated with toenail Pb concentration and the linear distance from the nearest Pb NEI site was inversely associated with toenail Pb concentration suggesting that those living in closer proximity to Pb NEI sites had greater Pb body burden. On the other hand, both Site and Emissions Hg IDW scores were inversely associated with toenail Hg, suggesting that residence farther from Hg IDW sites and exposure to smaller volumes of Hg NEI emissions were associated with higher toenail Hg concentration. Unexpectedly, we also found lower toenail concentrations of toxic (As, Hg) and essential metals (Mn, Se, and Zn) in Black participants compared to White participants.

Racial differences in toenail metal concentrations were corroborated by blood metal measurements of Hg, Mn, Pb, and Se collected in another GuLF sub-study (n = 1058) of which 723 of the participants were of the same age range, gender, and race as this study. Blood concentrations of As and Cr were not measured. Consistent with our toenail metal results, blood concentrations of Hg, Mn, and Se were also significantly lower in Black participants than in White participants adjusting for age, BMI, smoking history, passive smoke exposure, and employment status (Supplemental Fig. 3). And like our observations in the toenail, blood Pb concentrations were also not significantly different by race.

Compared to median concentrations reported in the National Health and Nutrition Examination Survey (NHANES) among White men of the same age range from the same period, median blood Hg concentrations from White GuLF Study participants were slightly higher, which may be reflective of higher locally caught seafood consumption in Gulf states (Sathiakumar et al., 2017), but this difference was not statistically significant (Supplemental Fig. 4). Blood concentrations of Mn, Pb, and Se from the GuLF Study were comparable to concentrations reported in NHANES (Supplemental Fig. 4).

The consistency of the observed racial disparity in Hg, Mn, Pb and Se body burden across matrices in the GuLF Study provides additional confidence for the reliability of the toenail metal biomarker, which has previously received pushback surrounding concerns about the lack of analytical standardization and potential for exogenous contamination (Gutiérrez-González et al., 2019). The reliability of our toenail Pb measurement is further supported by our finding that toenail Pb concentrations were inversely associated with the median year that structures were built within the residential census block group highlighting a well-documented relationship between older housing stock and the greater exposure to Pb through outdated exposure sources such as lead paint or pipes (Hauptman et al., 2023). Among the metals unmeasured in blood from the other GuLF sub-study, As has been validated as a biomarker of chronic exposure in the toenail (Martinez-Morata et al., 2023; Slotnick & Nriagu, 2006). No studies have been conducted to validate toenail Cr as a biomarker of Cr exposure, but our previous toenail reliability study found strong agreement in Cr measurements across triplicate toenail samples thus providing analytical confidence in this measurement (Lin et al., 2023).

We suspect that dietary or non-spill cleanup related occupational exposures, which were not well captured in the GuLF Study surveys, may explain some of the metal concentration disparities observed in this population. In particular, racial differences in toenail concentrations of Hg, Mn and Se may be attributable to dietary or nutritional differences between groups, as the predominant sources of exposure to these elements are through the diet (Martins et al., 2020; Rose et al., 2010). Since Hg in the toenail is largely comprised of methyl mercury (a common indicator of seafood intake) in non-occupational settings (Castro-González & Méndez-Armenta, 2008; Rose et al., 2010), it is possible that environmental Hg exposure from ambient industrial exposures is masked by seafood intake exposures in this coastal population. As such, the relationship between Hg NEI proximity metrics and toenail Hg concentration in this study may reflect income-related seafood intake differences in this group with those living in higher SES neighborhoods, farther away from NEI sites, also consuming more seafood.

Since the greatest sources of As exposure in the general population is through contaminated drinking water (Chung et al., 2014), it is possible that racial differences in toenail As concentration may be explained by differences in drinking water sources between White and Black participants related to their neighborhoods of residence. Further research focusing on drinking water As in this region is needed. On the other hand, Cr exposure is most often associated with occupational exposures or industrial processes (Sun et al., 2015; Wilbur et al., 2012). Studies with detailed occupational exposure data may be needed to explain racial differences in toenail Cr observed in this study.

Despite potential interferences from unmeasured dietary or occupational exposures, we found that greater residential proximity (distance and density) to Pb NEI sites was associated with higher toenail Pb concentration. The association persisted after adjusting for individual level education but the adjustment of census block group SES factors (percent of population below the poverty line and the median year that housing structures were built) in separate models resulted in appreciable attenuation. There was also no association between Emissions IDW score and toenail Pb concentration suggesting that the relationship between residential proximity to Pb-emitting NEI sites may be, in part, driven by the fact that neighborhoods closer to metal emitting sites are more likely to experience co-occurring exposures or other social stressors that may exacerbate their exposures to Pb. This phenomenon has been documented in previous studies in the U.S. showing that industry-adjacent neighborhoods typically receive fewer public works maintenance or remediation projects, have older housing stock, and have limited bargaining power to prevent toxic environmental exposures from ending up in their communities (Geron et al., 2022; Tyrrell et al., 2013).

Importantly, we found that within each income category, Black participants resided closer than White participants to metal-emitting NEI sites (Supplemental Fig. 1). Within the same income categories, Black participants were also more likely to reside in census block groups with a greater proportion of households below the poverty level, a trend that was observed across all 4 Gulf states (Supplemental Fig. 6). This is consistent with some of the known outcomes of historic redlining and other practices that promoted segregation. Previous reports from other regions in the U.S. have also found that individuals from minoritized racial and ethnic groups disproportionately live closer to industrial pollution (Mohai et al., 2009; Tyrrell et al., 2013).

In race-stratified analyses, it was clear that Black participants were driving the associations between toenail Pb and Hg concentrations and NEI determinants (Supplemental Fig. 2). A one-unit increase in Pb and Hg Site IDW score among Black participants was associated with a nearly 110% increase in toenail Pb concentration and 72% reduction in toenail Hg, respectively, while the association among White participants was smaller and not statistically significant (Supplemental Fig. 2). The same was observed for Hg Emissions IDW score. These findings suggest that Black participants may experience a disproportionate NEI metal exposure burden compared to White participants. However, the differences in NEI exposure burden could also be explained in part by differences in annual income by race. In this study, Black participants were disproportionately represented in the lowest income category. More than 48% (n = 92) of Black participants reported an annual income less than $20,000 and only 11% (n = 21) reported making more than $50,000 a year. In contrast, only 17% (n = 33) of White participants reported annual income below $20,000 and more than 46% reported making more than $50,000 a year.

A limitation of this work is the use of residential proximity from industry-reported air emissions sites and emissions volumes as the exposure metrics. There is no simple conversion of release quantity from NEI sites to the actual dose received by individuals since multiple processes can affect their fate and transport and determine how humans are eventually exposed to these pollutants (Brender et al., 2011; Huang & Batterman, 2000; Maantay, 2002). Furthermore, reporting to the NEI database is voluntary and designed for regulatory purposes. As such, data are limited to annual aggregate values and lack temporal or spatial variability. Another limitation is our use of socially constructed variables like race to delineate differences between groups, which may not perfectly capture differences in the way people experience environmental injustices. However, we do so in this study in efforts to describe the persistent effects of a long history of racial segregation and racist zoning laws in this region.

While limitations in emissions reporting and lack of detailed dietary/occupational information may have muddied the geospatial patterns of some of the ambient environmental exposures of NEI metals in this study, there remained a clear positive relationship between residential proximity to Pb NEI sites and Pb body burden. Given the attenuated associations after adjusting for neighborhood-level SES variables, it is possible that this relationship is driven, in part, by other socio-economic factors related to residence in more disadvantaged neighborhoods. Nonetheless, the consistency of the direction of Pb associations observed in this study is suggestive of a positive contribution from Pb-emitting industries on Pb body burden in neighboring communities. These findings highlight the importance of prioritizing continued Pb mitigation interventions in industry-proximal neighborhoods where residents can be co-exposed to Pb from multiple sources that may have detrimental consequences on health and well-being.

## 5. Conclusion

This study identified racial disparities in residential proximity to air metal emitting NEI sites in the US Gulf region and highlighted unexpectedly higher toenail concentrations of both toxic (As, Hg) and essential (Cr, Mn, Se) metals in Non-Hispanic White participants compared to Non-Hispanic Black participants. The significant associations between Pb-emitting NEI residential proximity and toenail Pb concentration highlight concerns about elevated Pb exposures in industry-proximate neighborhoods regardless of whether the exposures are coming directly from the facilities or from other factors related to industry adjacent residence. Interventions to reduce metal exposure in this population should focus particular attention on disparities by race and income. Further studies focusing on diet and occupation should be conducted to pinpoint – and mitigate where appropriate – the sources of the unexpected metal exposure disparities in this population.

## Figures and Tables

**Table 1:** Participant characteristics by self-reported race

	Total (n = 413) N (%)	NH White (n = 190) N (%)	NH Black (n = 191) N (%)
Age			
20–39	120 (29)	42 (22)	71 (37)
40–59	222 (54)	100 (52)	104 (54)
60–69	71 (17)	48 (25)	16 (9)
Highest educational attainment			
< High school	80 (19)	30 (16)	36 (19)
High school or equivalent	149 (36)	61 (32)	83 (43)
Some college	124 (30)	53 (27)	61 (32)
> College graduate	60 (15)	46 (24)	11 (6)
Annual Household Income			
< $20,000	130 (31)	33 (17)	91 (48)
$20,000 - $49,999	140 (34)	56 (29)	69 (35)
> $50,000	120 (29)	89 (46)	21 (11)
N/A	23 (6)	12 (6)	10 (5)
Smoking history			
Never	274 (66)	102 (54)	149 (78)
Former	139 (33)	88 (46)	42 (22)
Passive smoke exposure			
<30 mins/day	323 (78)	146 (77)	152 (80)
>30 mins/day	86 (21)	44 (23)	36 (19)
N/A	4 (1)	0 (0)	3 (1)
Employment status			
Working	253 (61)	136 (72)	96 (50)
Unemployed	123 (30)	31 (16)	85 (45)
Student/retired/other	37 (9)	23 (12)	10 (5)
State of residence			
AL	176 (43)	71 (38)	101 (53)
FL	55 (13)	33 (17)	15 (8)
LA	111 (27)	61 (32)	35 (18)
MS	70 (17)	24 (13)	39 (21)
Cumulative total average hydrocarbon (THC; ppm)			
Median (GSD)	91.6 (6.0)	85.4 (6.7)	102.2 (5.0)

## Data Availability

The data and code can be requested by email to the corresponding author.
